# An ultra-high-density protein microarray for high throughput single-tier serological detection of Lyme disease

**DOI:** 10.1038/s41598-020-75036-2

**Published:** 2020-10-22

**Authors:** Vasanth Jayaraman, Karthik Krishna, Yuanyuan Yang, Karenah J. Rajasekaran, Yuzheng Ou, Tianhao Wang, Kang Bei, Hari Krishnan Krishnamurthy, John J. Rajasekaran, Alex J. Rai, Daniel A. Green

**Affiliations:** 1 Vibrant Sciences LLC., San Carlos, CA USA; 2 Vibrant America LLC., San Carlos, CA USA; 3 Department of Pathology and Cell Biology, Columbia University Irving Medical Center, New York, NY USA

**Keywords:** Bacterial infection, Microbiology techniques, Assay systems, Lab-on-a-chip

## Abstract

Current serological immunoassays have inherent limitations for certain infectious diseases such as Lyme disease, a bacterial infection caused by *Borrelia burgdorferi* in North America. Here we report a novel method of manufacturing high-density multiplexed protein microarrays with the capacity to detect low levels of antibodies accurately from small blood volumes in a fully automated system. A panel of multiple serological markers for Lyme disease are measured using a protein microarray system, Lyme Immunochip, in a single step but interpreted adhering to the standard two-tiered testing algorithm (enzyme immunoassay followed by Western blot). Furthermore, an enhanced IgM assay was supplemented to improve the test’s detection sensitivity for early Lyme disease. With a training cohort (n = 40) and a blinded validation cohort (n = 90) acquired from CDC, the Lyme Immunochip identified a higher proportion of Lyme disease patients than the two-tiered testing (82.4% vs 70.6% in the training set, 66.7% vs 60.0% in the validation set, respectively). Additionally, the Immunochip improved sensitivity to 100% while having a lower specificity of 95.2% using a set of investigational antigens which are being further evaluated with a large cohort of blinded samples from the CDC and Columbia University. This universal microarray platform provides an unprecedented opportunity to resolve a broad range of issues with diagnostic tests, including multiplexing, workflow simplicity, and reduced turnaround time and cost.

## Introduction

Serological tests, which can be used to detect and characterize the antibody response to infection, are primarily formatted as immunoassays, including enzyme immunoassays (EIA), enzyme-linked immunosorbent assays (ELISA), Western blot (WB), immunoblot (IB), etc^1,2^. However, existing immunoassays have three fundamental limitations that have impeded their applications in disease diagnosis. First, conventional immunoassays are limited by their level of multiplexing, defined as the maximum number of antibodies that can be detected in a single run^3^. Second, most immunoassays have limited sensitivity for detection of antibodies at low levels, which can impede their utility in early stage diagnosis or in individuals with compromised immune systems with low antibody production^4^. Third, there is a lack of robust and flexible technology which can readily accept the addition of novel antigen biomarkers that have been validated for clinical use^3^.

Lyme disease, a bacterial infection caused primarily by *Borrelia burgdorferi* and a few cases by *Borrelia mayonni* in North America, is increasing in prevalence and public health importance, but its diagnosis can be impeded by the lack of accurate laboratory tests, particularly for early detection^5,6^. While early localized disease is usually accompanied by a characteristic erythema migrans (EM) rash, up to 20–30% of patients may lack this finding^7,8^ and if left untreated, bacteria can disseminate to cause nervous system involvement and/or cardiac conduction abnormalities^9,10^. Late disease can present as arthritis and/or a wide spectrum of nervous system manifestations^11^. In the United States, approximately 300,000 people are estimated to contract Lyme disease each year, but only 42,743 confirmed and probable cases of Lyme disease were reported to Centers for Disease Control and Prevention (CDC) in 2017, an increase of 17% from 2016^12^.

Assessment of the antibody response to *B. burgdorferi* infection has been the mainstay of laboratory confirmation of Lyme disease for over 20 years. A two-tiered serology algorithm, which utilizes a first-tier EIA followed by a second-tier WB or IB assay, was recommended in the United States and Europe(Fig. 2A)^13,14^. Contemporary EIAs have been developed using select recombinant proteins and/or select synthetic peptides from immunodominant regions within proteins that are specific to and conserved among the *B. burgdorferi *sensu lato (*sl*) complex members. WB analysis has been required to supplement positive EIA tests owing to its higher specificity and the ability to detect multiple protein antigens. However, blot-based testing is known to cause false positive results related to over-reading of weak bands in its gel matrix that affect quality of testing in Lyme disease, especially for IgM interpretation^15^. The two-tiered testing is inherently labor-intensive and uses subjective criteria for interpretation of results that may impact reproducibility with increased turnaround time and added complexity and cost^16^.

Previously, we developed a silicon-based peptide microarray platform utilizing a high-volume manufacturing and computational process derived from semiconductor industrial design. This novel technology has been validated in identification of highly predictive peptide antibody biomarkers in diagnosis and monitoring of Celiac disease^17,18^. In this study, we introduce a multiplex microarray of *Borrelia burgdorferi* recombinant antigens, wherein the proteins are physically separated from each other, to detect low levels of serological markers for Lyme disease from small blood volumes in a fully automated workflow(Fig. 2B). We explore here a novel testing methodology using a training cohort and blinded clinical samples from the CDC. With a functionalized high-density substrate, enhanced IgM assay, and a set of investigational markers, we aimed to improve both the analytical and clinical performance characteristics in Lyme disease testing.

## Materials and methods

### Patient samples

Two cohorts of serum samples acquired from CDC Lyme serum repository (LSR) were employed^19^. The training cohort contained 40 sera and the blinded validation cohort contained 90 sera. These serum samples were from Lyme disease positive patients at various stages with different sequelae, and negative controls including healthy controls and patients with other diseases. The composition of both cohorts is presented in Table 3. The informed consent and Institutional Review Board approval for testing these samples was granted during the establishment of LSR by CDC. Testing of reference sera was performed by laboratory personnel at Vibrant America Clinical Labs (CLIA and CAP accredited facility) without prior knowledge of the expected results in the same manner as clinical samples from Lyme disease patients. The CDC provided EIA results, detectable bands from WBs, WB interpretation by CDC criteria, and two-tiered testing results. CDC utilized VIDAS Lyme IgM and IgG polyvalent assay by bioMérieux, Inc for the EIA testing and MarDx Diagnostics IgM and IgG immunoblotting assays for the WB testing. The 142 healthy negative sera samples used to establish cut-off (CU) values was collected and tested under IRB exemption (work order #1-1098539-1) determined by the Western Institutional Review Board (WIRB) to use de-linked and de-identified human specimen and medical data for research purposes. The upper 97.5th percentile for each analyte was set to 10 CU and the upper 99th percentile was set to 20 CU (Supplemental Dataset). All methods used in this study were carried out in accordance with relevant guidelines and regulations.

### Wafer substrate preparation

**Figure 1:**
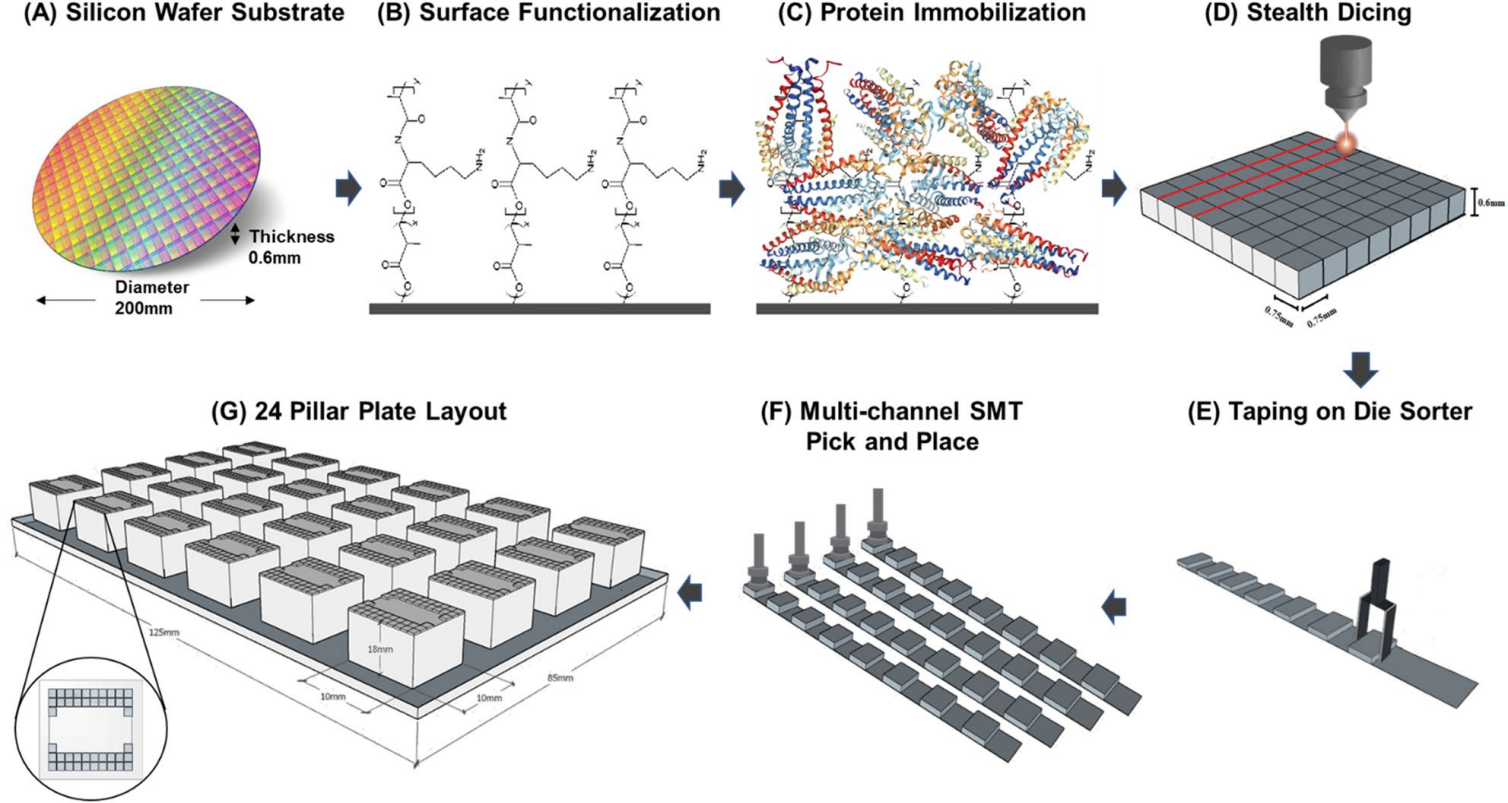
Microarray platform architecture. (**A**) The silicon wafer substrate is (**B**) functionalized with a copolymer composite of poly(lactic acid) and poly(L-lysine) and (**C**) subsequently immobilized with protein probes. OspC (PDB ID: 1GGQ) is shown as an example^20^. (**D**) Each probe-immobilized wafer substrate is diced into microchips using a stealth dicing process. (**E**) The diced microchips are picked and placed onto carrier tapes using a standard die sorting system. (**F**) The carrier tapes are loaded onto a high throughput surface mount technology (SMT) component placement system. (**G**) Individual microchips are picked and placed onto 24-pillar plates with each pillar containing a layout of 44 microchips.

Prime grade 300 mm silicon wafers with p-type boron, (1 0 0) orientation, resistivity of 1 to 5 Ω cm^−1^, and 725 μm thickness were deposited with 100 nm thermal oxide by dry oxidation at 1,000 °C in a furnace under pure oxygen atmosphere for two hours. Wafers were copiously washed with deionized water for five minutes and spin coated with a solution containing 1% (vol/vol) of 3-aminopropyltriethoxysilane (APTES) in N-methylpyrrolidone (NMP) and left at room temperature for 15 min. Curing of the wafers was done at 120 °C for 60 min under N_2_ atmosphere and humidity-controlled environment. A copolymer solution containing poly(L-lysine) and poly(lactic acid) was coated on the wafer and reacted for 24 h to generate a high binding surface capable of immobilizing proteins via passive adsorption through hydrophobic interactions with lysine polymer and covalent coupling with the lactic acid polymer (structures shown in Fig. 1B). The functionalized wafers were normally used within one week of preparation.

### Protein attachment

The following antigens were included in the panel: *Borrelia burgdorferi* VlsE1, *Borrelia burgdorferi* p18, *Borrelia burgdorferi* p23, *Borrelia burgdorferi* p28, *Borrelia burgdorferi* p30, *Borrelia burgdorferi* p31, *Borrelia burgdorferi* p34, *Borrelia burgdorferi* p39, *Borrelia burgdorferi* p41, *Borrelia burgdorferi* p45, *Borrelia burgdorferi* p58, *Borrelia burgdorferi* p66, *Borrelia burgdorferi* p93. Figure 1C shows *Borrelia burgdorferi* p23 as an example for the protein immobilization process. The recombinant antigens were expressed in E. coli bacterial cells using full length cDNA coding for the respective antigens fused with a hexa histidine purification tag. The capture antigens were incubated on the wafer at a concentration of 1.0 μg/ml and reacted for 24 h at 4 °C. Excess unbound antigens were removed by extensive washing with aqueous phosphate buffer and the unreacted substrate was quenched with a blocking solution containing BSA and glycine. Each wafer was patterned with a unique identifier to classify the antigens attached.

### Pillar plate assembly

The pillar plate assembly process is illustrated in Fig. 1d–g. The individual wafers for each antigen were then diced into 0.7 × 0.7 mm^2^ microchips using a stealth dicing process, as previously described^21^. The diced wafers were picked and placed onto individual carrier tapes using a standard die sorting system. The carrier tapes were loaded onto a high-throughput surface mount technology (SMT) component placement system. The microchips were then picked and placed onto 24-pillar plates with each pillar containing a layout of 44 microchips.

### Immunochip assay

Serum samples were probed using 1:20 dilution on the pillar plate and reacted for one hour at room temperature as previously described^21^. The plate was then washed with Tris-buffered saline with Tween 20 (TBST) (Amresco) buffer 3 × 5 min each. The plate was incubated with the secondary antibody (1:2000 dilution of Goat Anti-Human IgG HRP and Goat Anti-Human IgM HRP individually) for one hour at room temperature. The plates were washed with TBST buffer followed by washing with DI Water. The plates were finally dried completely before adding chemiluminescent substrate (Lumi-Phos HRP from Lumigen), per manufacturer recommendations and scanned for five minutes on a standard Chemiluminescence Imager. For the Enhanced IgM Assay, the serum was pre reacted with Goat anti-human IgG Fc fragment prior to the remaining assay steps to increase the sensitivity of IgM detection.

### Data analysis

The generated images are analyzed using the in-house reporter software as described previously^21^. Briefly, the chemiluminescent signals are converted into intensity plots which was compared to the thresholds established for each antigen to determine sero-positivity.

## Results

### Lyme immunochip platform

**Figure 2:**
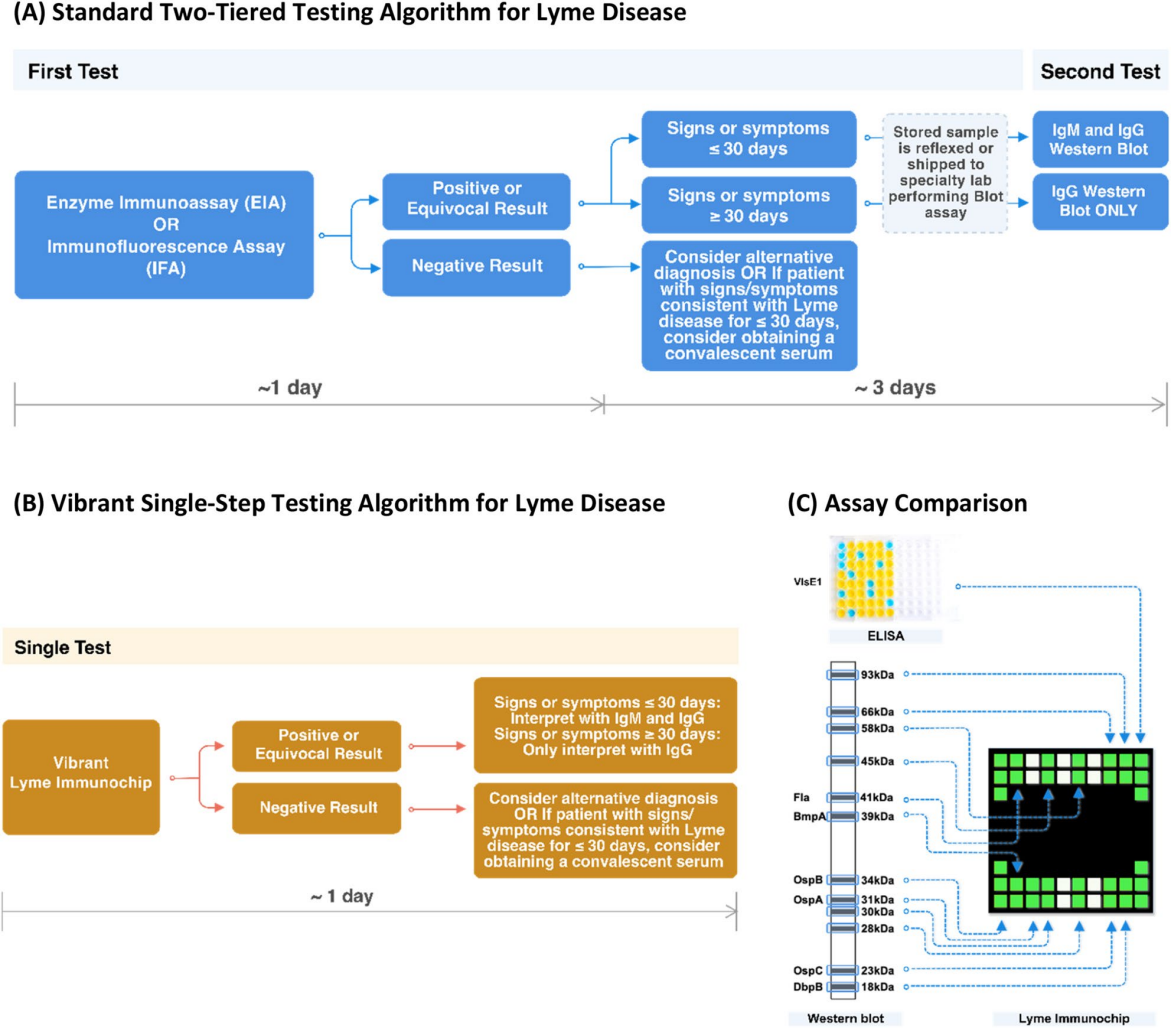
(**A**) Standard two-tiered testing algorithm recommended by CDC. (**B**) Vibrant Lyme Immunochip testing algorithm. (**C**) Different bands on a typical Western blot / Immunoblot are tested one protein per microchip using multiple microchips per pillar.

The main components of the Lyme Immunochip platform include multiple silicon-based 0.70 × 0.70 mm^2^ microchips that are laser diced from antigen-immobilized wafers, a customized 24-well compatible plate containing 24 pillars, each containing 44 microchips that are picked and placed into a multiplex microarray assembly, and a high-resolution imager capable of simultaneously detecting chemiluminescent signals from labeled antigen–antibody reactions at each microchip throughout the multiplex microarray (Fig. 1). Each chip can be considered analogous to an individual band in a Western blot; however, the proteins are physically separated eliminating cross-reactive issues seen in blot-based assays for proteins with similar mass (Fig. 2C)^22,23^.

### Multiplex antibody detection

Detection of multiplex antibodies is based on a chemiluminescent immunoassay. In contrast to two-tiered testing by EIA and WB, the multiplex test on Lyme Immunochip requires only 25.0 μL of serum specimen. The entire process including sample dilution, multi-step incubation, and multi-solution washing is programmed into liquid handlers. Coupled with automatic detection of analytes and data acquisition, the Lyme Immunochip has the capacity to simultaneously assay 192 individual specimens within 2 h. An in-house reporter software is designed to extract the raw chemiluminescent signals for each probe and convert them into intensity plots after quantile normalization, background, and spatial correction. Compared with traditional two-tiered testing (Fig. 2A), automatic detection of multiple antigens in this high-throughput manner (Fig. 2B) has the potential to dramatically shorten turnaround time, reduce the cost of labor and instrument, and eliminate the need of manual handling and subjective interpretation of the WB or IB test results. All antibodies are detected in a single run, but the results are interpreted according to the standard two-tiered algorithm (Table 1). Here we chose VlsE1 (variable major protein like sequence expressed), which is one of the most commonly targeted proteins in the EIA test, as the first tier. The second tier employs the same antigens and interpretation rule as recommended by the Second National Conference on Serologic Diagnosis of Lyme disease—an IgM immunoblot is considered positive if two of the three bands are present and IgG immunoblot is considered positive if five of the 10 bands are present (Table 1). Recently, the CDC has suggested a second EIA in place of WB as acceptable alternatives for the serologic diagnosis of Lyme disease which is expected to accelerate the move to non-blot based testing methods^19^. The sensitivity and specificities of the individual antigens are shown in Supplemental Table S1.

**Table 1:** Composition of the Vibrant’s Lyme Immunochip and interpretation with the standard two-tiered testing algorithm.

Species	Antigen	Antibody
**1st Tier**		
*B. burgdorferi*	VIsE1	IgM/IgG
**2nd Tier**		
*B. burgdorferi*	p23(OspC), p39(BmpA), p41(Fla)	IgM
*B. burgdorferi*	p18(DbpB), p23(OspC), p28(Oms28), p30, p39(BmpA), p41(Fla), p45, p58(OppA-2), p66(Oms66), p93	IgG
**For research purpose**		
*B. burgdorferi*	p18(DbpB), p28(Oms28), p30, p31(OspA), p34(OspB), p45, p58(OppA-2), p66(Oms66), p93	IgM
*B. burgdorferi*	p31(OspA), p34(OspB)	IgG

### Analytical performance

We evaluated the analytical performance characteristics of the Lyme Immunochip for the following parameters: precision (repeatability/reproducibility), analytical sensitivity, and analytical specificity. Simple precision (repeatability) was determined by running three samples over the assay measuring range 10 times within the same run. Complex precision (reproducibility) was determined by assaying two replicates of three samples twice daily over five days. In addition, one positive and one negative control were included in each run. The coefficients of variation for repeatability and reproducibility considering all antigens are shown in Table 2. The analytical sensitivity was determined by testing protein-free serum matrix samples with 20 replicates per run. The mean and standard deviation of blank was used to calculate limit of blank (LoB). The LoB and standard deviation of 6 low concentration samples was used to calculate the limit of detection (LoD), as shown in Table 2. Furthermore, an interfering substance study was conducted to evaluate the potential interference of specific endogenous and exogenous substances with Lyme Immunochip. The interfering substances with levels tested include 40 mg/dl Bilirubin, 500 mg/dl Cholesterol, 1000 mg/dl Triglycerides, 200 mg/dl Hemoglobin, 120 g/L Albumin, and 3000 U/L Heparin. No interference was observed with any of the substances tested at the stated levels.

**Table 2:** Analytical performance of the Lyme Immunochip.

Antibody	Coefficient of variation for repeatability (%)			Coefficient of variation for reproducibility (%)			Analytical sensitivity	
	Sample 1	Sample 2	Sample 3	Sample 1	Sample 2	Sample 3	LoB (CU)	LoD (CU)
*B. Burgdorferi* Vlse1 IgM	2.87	2.33	5.30	6.95	3.77	1.77	0.3	0.8
*B. Burgdorferi* Vlse1 IgG	4.14	1.92	1.93	5.28	4.70	1.26	0.3	0.7
*B. Burgdorferi* p23 IgM	4.79	2.11	3.84	1.94	1.57	2.94	0.3	0.8
*B. Burgdorferi* p39 IgM	3.39	3.72	4.11	3.64	1.73	2.44	0.4	0.8
*B. Burgdorferi* p41 IgM	3.93	2.93	2.65	1.73	6.34	1.93	0.1	0.7
*B. Burgdorferi* p18 IgG	4.04	1.85	2.63	2.09	2.05	2.04	0.4	0.8
*B. Burgdorferi* p23 IgG	3.10	9.52	2.21	4.72	2.03	1.72	0.3	0.6
*B. Burgdorferi* p28 IgG	1.56	5.08	1.56	2.15	3.92	7.14	0.5	0.8
*B. Burgdorferi* p30 IgG	1.53	4.42	3.59	2.73	3.92	8.13	0.4	0.6
*B. Burgdorferi* p39 IgG	1.26	8.59	2.59	2.37	2.32	2.89	0.3	0.8
*B. Burgdorferi* p41 IgG	1.91	1.68	1.29	2.33	5.67	2.28	0.2	0.5
*B. Burgdorferi* p45 IgG	1.49	1.62	2.29	4.69	4.40	3.65	0.4	0.5
*B. Burgdorferi* p58 IgG	1.57	2.34	4.58	5.30	4.43	1.47	0.2	0.7
*B. Burgdorferi* p66 IgG	4.15	2.18	8.69	4.80	2.67	8.37	0.1	0.6
*B. Burgdorferi* p93 IgG	1.82	4.00	1.75	6.44	1.61	4.61	0.2	0.7

### Training of the Lyme Immunochip with clinical samples

**Figure 3:**
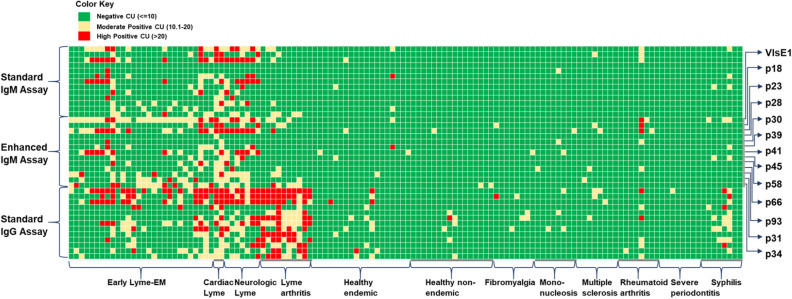
Heat map of antibody-binding intensities in the training and validation sets of clinical samples. A positivity cutoff for each marker was defined as greater than 10 chemiluminescent units (yellow or red). Samples additionally captured in the Enhanced IgM assay have been compared side by side in the supplemental material. All three assays detect antibodies to the markers presented on the right y-axis of the heat map. These markers are not repeatedly labeled for the Standard IgM Assay and Standard IgG Assay. Chemiluminescent units for each sample are also shown in the Supplemental Data File.

A training set of 40 samples was acquired from the CDC’s Lyme Serum Repository^24^ (LSR) and used to optimize the cut-offs of the Lyme Immunochip. Of the 40 samples, 17 samples were from patients diagnosed with Lyme disease (clinically characterized borreliosis stratified by disease stage) and 23 samples were from control individuals (disease controls and negative controls), as shown in Table 3. A positivity cutoff for each marker was defined as greater than 10 chemiluminescent units (CU). The cut-off was established by testing an external set of 142 healthy negative serum samples from endemic and non-endemic regions. The maximum signal from the negative samples plus two standard deviations was used to establish the assay cut-off and set at 10 CU for all antigens. For early Lyme patients at acute and convalescent phases, the standard IgM and IgG assays (as shown in Fig. 3) provided 60.0% sensitivity on the Lyme Immunochip, compared with the standard two-tiered testing’s sensitivity of 50.0%. All late Lyme patients tested positive (100% sensitivity) and none of the disease or healthy controls tested positive (100% specificity). The sensitivity and specificity levels demonstrated by the Lyme Immunochip were higher than those seen with standard FDA-approved two-tiered testing.

**Table 3:** Clinical performance of the Lyme Immunochip in the training and validation cohorts.

Cohort and subject type	n	Vibrant Lyme Immunochip				Standard two-tiered testing	
		Enhanced IgM		Standard			
		Sensitivity (%)	Specificity (%)	Sensitivity (%)	Specificity (%)	Sensitivity (%)	Specificity (%)
Training cohort	40						
Early Lyme disease—acute/convalescent	10	70.0		60.0		50.0	
Neurologic Lyme	3	100.0		100.0		100.0	
Lyme arthritis	4	100.0		100.0		100.0	
Total positive:	17	82.4		76.5		70.6	
Fibromyalgia	2		100.0		100.0		100.0
Rheumatoid arthritis	2		100.0		100.0		100.0
Mononucleosis	2		100.0		100.0		100.0
Syphilis	2		100.0		100.0		100.0
Multiple sclerosis	2		100.0		100.0		100.0
Severe periodontitis	2		100.0		100.0		100.0
Healthy endemic	7		100.0		100.0		100.0
Healthy non-endemic	4		100.0		100.0		100.0
Total negative:	23		100.0		100.0		100.0
Validation cohort	90						
Early Lyme disease—acute/convalescent	18	50.0		38.9		38.9	
Neurologic Lyme	4	75.0		75.0		75.0	
Cardiac Lyme	2	100.0		100.0		100.0	
Lyme arthritis	6	100.0		100.0		100.0	
Total positive:	30	66.7		60.0		60.0	
Fibromyalgia	6		100.0		100.0		100.0
Rheumatoid arthritis	6		100.0		100.0		83.3
Mononucleosis	6		100.0		100.0		100.0
Syphilis	6		66.7		66.7		83.3
Multiple sclerosis	6		100.0		100.0		100.0
Severe periodontitis	6		100.0		100.0		100.0
Healthy endemic	12		100.0		100.0		100.0
Healthy non-endemic	12		100.0		100.0		100.0
Total Negative	60		96.7		96.7		96.7
Complete panel with established markers	130	72.3	97.6	66.0	97.6	63.8	97.6
Complete panel with investigational markers	130	100.0	95.2	74.5	94.0	NA	NA

### Enhanced IgM assay

To further improve the accuracy for diagnosis of early Lyme disease, an in-house enhanced IgM assay was developed and utilized. IgM antibodies are the first type of antibody produced by the immune system in response to an infection. lgM antibodies only comprise 5% to 10% of all the antibodies present in circulation, whereas lgG antibodies, which are the most abundant immunoglobulin, comprise about 75% to 80%^25^. In order to enhance sensitivity and specificity of the IgM assay, we reduced assay interference by removing a major portion of the abundant lgG antibodies and other non-specific proteins from the serum while detecting the IgM antibodies. A purified goat anti-human (GAH) IgG Fc fragment was incubated with serum for the removal of human IgG prior to testing for specific IgM antibodies. In the training set, the enhanced IgM assay further improved the sensitivity to 70.0% for early Lyme patients while the performance was equivalent for the rest of the disease/control samples as shown in Table 3. Samples captured by enhanced IgM but not standard IgM are shown in Supplementary Figure S1. There were also no additional false positives with the enhanced IgM assay as compared to the standard IgM assay as seen in Fig. 3.

### Blinded clinical sample validation

To validate the clinical performance of the Lyme Immunochip, a cohort of blinded samples (N = 90) acquired from the CDC LSR was tested and interpreted by criteria achieved via the training set. After incorporating the enhanced IgM assay, the single-tier Immunochip was compared with standard two-tiered serologic testing. Whereas the Immunochip identified 9 out of 18 early Lyme disease patients (50.0%) at acute and convalescent phases, the standard two-tiered testing identified only 7 of them (38.9%). The clinical performance by both methods were identical towards later-stage Lyme disease, including neurologic Lyme disease (75.0%), cardiac Lyme disease (100.0%), and Lyme arthritis (100.0%). The specificity levels achieved by this single-tier assay was identical to that of the two-tiered testing (96.7%). The heat map in Fig. 3 shows antibody-binding intensity data differentiating samples seropositive for Lyme disease from healthy controls and disease controls. The enhanced IgM assay was able to recognize two early Lyme disease samples at convalescent phase that were missed by the standard IgM assay. Cardiac Lyme disease, neurologic Lyme disease and Lyme arthritis exhibited higher-intensity binding by the IgG assay as compared to the IgM assays (Fig. 3). The Immunochip demonstrated sensitivity and specificity of 66.7% and 96.7% compared with those of CDC’s 60.0% and 96.7% in the blinded validation cohort. A set of investigational markers including p31 and p34 (Table 1) was also separately evaluated with interpretative criteria requiring binding of the first-tier antigen (VlsE1) plus any two second-tier antigens. While these investigational markers demonstrated higher sensitivity (100%) than the standard two-tiered antigen panel, they were also associated with lower specificity (95.2%) and require further evaluation with expanded sample sets. As shown in Fig. 3, the improvement in sensitivity was almost exclusively due to the inclusion of p31 and p34 in the enhanced IgM assay, which was particularly useful for detecting patients with early Lyme Disease.

## Discussion

Here, we present the first ultra-high-density microarray platform to rapidly and accurately detect a panel of well-characterized Lyme disease serological markers. The Lyme Immunochip applies the standard two-tiered testing algorithm criteria, but all markers are detected in a single run, which substantially improves turnaround time, reduces the cost of labor and instrument, and eliminates the need of manual handling and subjective interpretation of the WB or IB test results.

Multiplexing of proteins has been previously performed using spotted arrays or WB/IB^26^. Although some of these platforms have been used in diagnostic testing and life science research, they have important limitations. Protein spraying/spotting techniques are inconsistent due to variation in the volume of reagents taken up and dispensed owing to differences in reagent viscosity^27^. “Missing spots” or “merged spots” result from uneven surfaces, which hinders consistent contact between the printers/spotters and the chip surface^28^. Blot-based techniques additionally require skilled technologists to interpret bands, which leads to variability in interpretation, especially for weak bands^29^. Although automated readers have been reported for immunoblots, manufacturing technology of existing multiplex platforms are not capable of high throughput testing and mass production, which impacts their clinical utility at scale^30,31^.

To address these issues, we fundamentally innovated on the technology at every stage, including chip manufacturing, surface functionalization, array assembly, analyte detection, and data acquisition in the Vibrant Protein Microarray platform. The Vibrant pillar plate can incorporate 100 probe-immobilized microchips (in its current configuration) at each pillar, i.e., 100 types of biomarkers can potentially be detected for 24 samples in one pillar plate within a 1.0 cm^2^ pillar. Further compaction of the microchip can increase the number of biomarkers on each pillar plate and enhance the total throughput. For example, reducing the microchip size from 0.70 × 0.70 mm^2^ to 0.35 × 0.35 mm^2^, which is feasible (data not shown), would quadruple the capacity to 400 probes per pillar. Importantly, given that *Ixodes* ticks transmit other human pathogens such as *Anaplasma*, *Babesia,* and *Ehrlichia,* this ultra-high level of multiplexing allows for the further development of panels that can simultaneously assess for multiple tickborne diseases from a single, low-volume blood sample, with the added flexibility to incorporate emerging pathogens that become more prevalent over time.

Clinically the Lyme Immunochip showed higher overall sensitivity (72.3%) than standard two-tier testing (63.8%) (Table 3). VlsE1 and OspC were the two most sensitive markers observed on our Lyme Immunochip (a complete list of individual *B. burgdorferi* antigens’ clinical performance is shown in Figure S1). VlsE1 is composed of alternating variable (VR1 to VR6) and invariable (IR1 to IR6) regions, among which IR6 has been identified as an immunodominant epitope^32^. OspC is the second most-commonly targeted antigen in diagnostic testing and has an important role in transmission of Lyme disease-associated *Borrelia* from the tick vector to the mammalian host. OspC is available for immune stimulation sooner than VlsE because it is expressed on the surface of *B. burgdorferi* before infection^33^.

These sensitivity gains did not come at the cost of specificity, which was equivalently high for both the Lyme Immunochip and standard two-tier testing (97.6% each). Test specificity is a paramount concern for Lyme disease assays to mitigate the risk of false positives in low prevalence settings, and we therefore designed the Lyme Immunochip to utilize the same highly specific markers and stringent interpretation criteria used in the standard two-tier algorithm. The most specific markers in the panel were p58 and p39. The function of p58 is suggested to be involved in the transmembrane transport of solutes and but it shares a functional domain usually found in periplasmic oligopeptide-binding proteins^34^. *B. burgdorferi* basic membrane protein A (BmpA), also known as p39, localizes to the bacterium's outer membrane and binds to laminin in the host’s extracellular matrix^35^. The high specificity observed for these two markers is in accordance with previous reports but their exact functions are still elusive^36,37^. Recently, CDC recommended a modified two-EIA algorithm as an alternative to Western blot confirmation. While this approach allows for the accurate diagnosis of most straightforward cases with lower cost and faster turnaround time than Western blot, knowledge of which bands/antigens are reactive can aid in the interpretation of challenging cases, help differentiate new vs. past infections, and also measure the expansion of immune response over time^38^. The Lyme Immunochip can provide the same detailed information as Western blot, but with a fast turnaround time like that offered by a two-EIA approach. The test report can therefore include detailed antigen reactivity along with an overall positive/negative interpretation according to CDC guidelines and duration of symptoms (Fig. 2).

As the known repertoire of *B. burgdorferi* surface and secreted proteins expands over time, additional markers can be easily incorporated into the platform, enabling further performance improvements. In a previous study, a ten-antigen panel was selected from 62 *B. burgdorferi* surface proteins and synthetic peptides, which yielded 87.5% sensitivity for early Lyme disease by scoring the presence of any two markers as a positive result^39^. Based on a similar criterion, our preliminary study using our current protein microarray with all investigational antigen markers (as listed in Table 1) raised the sensitivity to 100.0% while lowering specificity to 95.2% for the two cohorts altogether; further validation with a large blinded cohort is underway. In addition, we are actively investigating previously unappreciated epitopes of highly immunogenic antigens that are targeted by the immune system using a peptide microarray. Generating an ultra-high-density peptide microarray with these additional epitopes may further improve clinical performance, identify disease subsets, and also provide fundamental insights into the pathogenesis of Lyme disease, including post-treatment Lyme disease syndrome.

In conclusion, we demonstrate that the Lyme Immunochip is a valid alternative to standard two-tiered testing, with the capability to characterize the antibody profile of Lyme disease patients with enhanced accuracy and reduced turnaround time. The advances in semiconductor methods and the generation of high-throughput microarrays for creating antigen diversity provides the potential to improve the diagnostic accuracy of immunoassays. The novel protein microarray method described herein provides three major advantages over existing testing methodologies: (1) an ultra-high-density array surface, (2) higher reproducibility, and (3) improved throughput. This platform permits simultaneous detection of a large number of candidate biomarkers from low volume samples with reduced cost and turnaround time, allowing for both multiplex diagnostic testing as well as biomarker discovery and validation. This ultra-high-density microarray has the potential to be an accurate, rapid, economic, and universal diagnostic testing platform.

## Supplementary information

Supplementary Information 1.

Supplementary Information 2.
