# Supplementary material for: An ultra-high-density protein microarray for high throughput single-tier serological detection of Lyme disease
Source: Sci Rep. 2020 Oct 22;10:18085. doi: 10.1038/s41598-020-75036-2 (PMC7581523; doi:10.1038/s41598-020-75036-2)
Supplement: Supplementary file 2 — Supplementary Information 2. [file 41598_2020_75036_MOESM2_ESM.docx]

**Supplemental Information**

**An Ultra-High-Density Protein Microarray for High Throughput Single-Tier Serological Detection of Lyme Disease**

Vasanth Jayaraman^1+^, Karthik Krishna^1^, Yuanyuan Yang^2^, Karenah J. Rajasekaran^2^, Yuzheng Ou^2^, Tianhao Wang^1^, Kang Bei^1^, Hari Krishnan Krishnamurthy^1^*^+^, John J. Rajasekaran^1^, Alex J. Rai^3^, Daniel A. Green^3*^

*^1^Vibrant Sciences LLC., San Carlos, CA, USA*

*^2^Vibrant America LLC., San Carlos, CA, USA*

*^3^Columbia University Irving Medical Center, New York, New York, USA*

**Table S1.** Comparison of the sensitivity and specificity of individual *B. burgdorferi* antigens

| **Antigen** |  | **Sensitivity for Lyme Disease** | | | |  | **Specificity for Lyme Disease** | | |
| --- | --- | --- | --- | --- | --- | --- | --- | --- | --- |
|  |  | **Stage 1** | **Stage 2** | **Stage 3** | **Overall** |  | **Disease Control** | **Healthy Control** | **Overall** |
| **Standard IgM Assay** |  |  |  |  |  |  |  |  |  |
| *B. burgdorferi* VlsE1 IgM |  | 39.3% | 100.0% | 20.0% | 46.8% |  | 97.1% | 95.8% | 96.4% |
| *B. burgdorferi* p18(DbpB) IgM |  | 10.7% | 44.4% | 10.0% | 17.0% |  | 100.0% | 95.8% | 97.6% |
| *B. burgdorferi* p23(OspC) IgM |  | 42.9% | 100.0% | 10.0% | 46.8% |  | 97.1% | 97.9% | 97.6% |
| *B. burgdorferi* p28(Oms28) IgM |  | 0.0% | 0.0% | 0.0% | 0.0% |  | 100.0% | 100.0% | 100.0% |
| *B. burgdorferi* p30 IgM |  | 3.6% | 0.0% | 0.0% | 2.1% |  | 100.0% | 97.9% | 98.8% |
| *B. burgdorferi* p39(BmpA) IgM |  | 21.4% | 33.3% | 0.0% | 19.1% |  | 97.1% | 97.9% | 97.6% |
| *B. burgdorferi* p41(Fla) IgM |  | 28.6% | 88.9% | 0.0% | 34.0% |  | 100.0% | 100.0% | 100.0% |
| *B. burgdorferi* p45 IgM |  | 10.7% | 22.2% | 0.0% | 10.6% |  | 97.1% | 95.8% | 96.4% |
| *B. burgdorferi* p58(OppA-2) IgM |  | 14.3% | 33.3% | 0.0% | 14.9% |  | 100.0% | 100.0% | 100.0% |
| *B. burgdorferi* p66(Oms66) IgM |  | 7.1% | 33.3% | 0.0% | 10.6% |  | 100.0% | 100.0% | 100.0% |
| *B. burgdorferi* p93 IgM |  | 14.3% | 66.7% | 10.0% | 23.4% |  | 100.0% | 100.0% | 100.0% |
| *B. burgdorferi* p31 IgM |  | 21.4% | 22.2% | 0.0% | 17.0% |  | 97.1% | 100.0% | 98.8% |
| *B. burgdorferi* p34 IgM |  | 25.0% | 33.3% | 40.0% | 29.8% |  | 100.0% | 100.0% | 100.0% |
| **Enhanced IgM Assay** |  |  |  |  |  |  |  |  |  |
| *B. burgdorferi* VlsE1 IgM |  | 96.4% | 100.0% | 30.0% | 83.0% |  | 97.1% | 89.6% | 92.8% |
| *B. burgdorferi* p18(DbpB) IgM |  | 10.7% | 44.4% | 20.0% | 19.1% |  | 100.0% | 93.8% | 96.4% |
| *B. burgdorferi* p23(OspC) IgM |  | 57.1% | 100.0% | 10.0% | 55.3% |  | 94.3% | 91.7% | 92.8% |
| *B. burgdorferi* p28(Oms28) IgM |  | 0.0% | 11.1% | 0.0% | 2.1% |  | 100.0% | 100.0% | 100.0% |
| *B. burgdorferi* p30 IgM |  | 3.6% | 11.1% | 0.0% | 4.3% |  | 100.0% | 95.8% | 97.6% |
| *B. burgdorferi* p39(BmpA) IgM |  | 21.4% | 33.3% | 0.0% | 19.1% |  | 97.1% | 97.9% | 97.6% |
| *B. burgdorferi* p41(Fla) IgM |  | 39.3% | 88.9% | 0.0% | 40.4% |  | 94.3% | 95.8% | 95.2% |
| *B. burgdorferi* p45 IgM |  | 10.7% | 44.4% | 0.0% | 14.9% |  | 97.1% | 95.8% | 96.4% |
| *B. burgdorferi* p58(OppA-2) IgM |  | 21.4% | 44.4% | 0.0% | 21.3% |  | 100.0% | 100.0% | 100.0% |
| *B. burgdorferi* p66(Oms66) IgM |  | 10.7% | 33.3% | 10.0% | 14.9% |  | 100.0% | 93.8% | 96.4% |
| *B. burgdorferi* p93 IgM |  | 32.1% | 77.8% | 20.0% | 38.3% |  | 97.1% | 100.0% | 98.8% |
| *B. burgdorferi* p31 IgM |  | 39.3% | 55.6% | 10.0% | 36.2% |  | 100.0% | 97.9% | 98.8% |
| *B. burgdorferi* p34 IgM |  | 57.1% | 44.4% | 50.0% | 53.2% |  | 94.3% | 100.0% | 97.6% |
| **IgG Assay** |  |  |  |  |  |  |  |  |  |
| *B. burgdorferi* VlsE1 IgG |  | 71.4% | 100.0% | 100.0% | 83.0% |  | 97.1% | 85.4% | 90.4% |
| *B. burgdorferi* p18(DbpB) IgG |  | 39.3% | 100.0% | 100.0% | 63.8% |  | 97.1% | 91.7% | 94.0% |
| *B. burgdorferi* p23(OspC) IgG |  | 53.6% | 100.0% | 90.0% | 70.2% |  | 94.3% | 95.8% | 95.2% |
| *B. burgdorferi* p28(Oms28) IgG |  | 14.3% | 22.2% | 20.0% | 17.0% |  | 97.1% | 93.8% | 95.2% |
| *B. burgdorferi* p30 IgG |  | 0.0% | 22.2% | 80.0% | 21.3% |  | 97.1% | 93.8% | 95.2% |
| *B. burgdorferi* p39(BmpA) IgG |  | 10.7% | 66.7% | 100.0% | 40.4% |  | 88.6% | 85.4% | 86.7% |
| *B. burgdorferi* p41(Fla) IgG |  | 42.9% | 88.9% | 100.0% | 63.8% |  | 94.3% | 95.8% | 95.2% |
| *B. burgdorferi* p45 IgG |  | 28.6% | 55.6% | 60.0% | 40.4% |  | 91.4% | 87.5% | 89.2% |
| *B. burgdorferi* p58(OppA-2) IgG |  | 17.9% | 44.4% | 80.0% | 36.2% |  | 97.1% | 100.0% | 98.8% |
| *B. burgdorferi* p66(Oms66) IgG |  | 10.7% | 33.3% | 90.0% | 31.9% |  | 97.1% | 91.7% | 94.0% |
| *B. burgdorferi* p93 IgG |  | 14.3% | 55.6% | 70.0% | 34.0% |  | 97.1% | 95.8% | 96.4% |
| *B. burgdorferi* p31 IgG |  | 28.6% | 33.3% | 60.0% | 36.2% |  | 94.3% | 95.8% | 95.2% |
| *B. burgdorferi* p34 IgG |  | 21.4% | 55.6% | 70.0% | 38.3% |  | 91.4% | 97.9% | 95.2% |


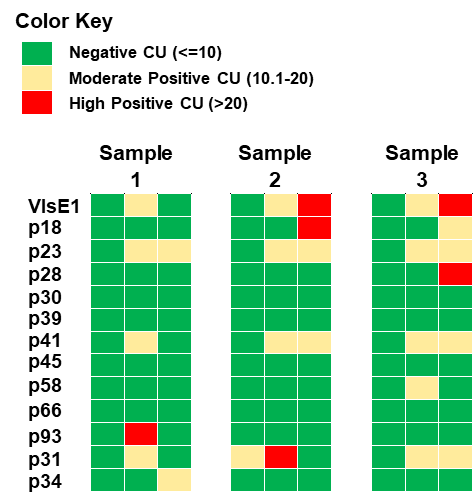


**Figure S1**. Heat map of antibody-binding intensities of three Early Lyme samples that were missed by the standard IgM assay but picked by the enhanced IgM assay in the training and validation sets. For each sample, the three columns from left to right represent results by standard IgM assay, enhanced IgM assay, and standard IgG assay, individually. All three assays detect antibodies to the markers presented on the left y-axis of the heat map. Intensity cutoff is 10 CU shown in yellow.
